# Complete mitochondrial genome of the composting worm *Dendrobaena veneta* (Clitellata: Oligochaeta, Lumbricidae)

**DOI:** 10.1080/23802359.2023.2265177

**Published:** 2023-10-13

**Authors:** Csaba Csuzdi, Jachoon Koo, Nak jung Choi, Tímea Szederjesi, Yong Hong

**Affiliations:** a Department of Zoology, Eszterházy Károly Catholic University, Eger, Hungary; b Division of Science Education and Institute of Fusion Science, College of Education, Jeonbuk National University, Jeonju, Korea; c Crop Foundation Research Division, National Institute of Crop Science, Korea; d Department of Systematic Zoology, Eötvös Loránd University, Budapest, Hungary; e Department of Agricultural Biology, College of Agriculture and Life Sciences, Jeonbuk National University, Jeonju, Korea

**Keywords:** *Dendrobaena veneta*, mitochondrial genome, Lumbricidae, phylogeny

## Abstract

*Dendrobaena veneta* (Rosa, 1886) is widely distributed all over Europe due to its use as compost worm. The specimen presented here was collected in Tiranë district, Albania. Currently, only two species’ complete or nearly complete mitochondrial genome (mitogenome) sequences have been reported in the genus *Dendrobaena*; *D. octaedra* (Savigny, 1826) and *D. tellermanica* Perel, 1966. In this study, the complete mitogenome of *D. veneta* was sequenced, assembled, and annotated. The mitogenome of *D. veneta* is a circular DNA molecule, consisting of 15,475 bp with an A + T content of 61.2%. It contains 13 protein-coding genes, 2 ribosomal RNA genes, 22 transfer RNA genes, and 1 non-coding region (control region). Phylogenetic analysis showed that *D. veneta* is clustered with the other two *Dendrobaena* species in the well-supported family Lumbricidae.

## Introduction

**Figure 1.**
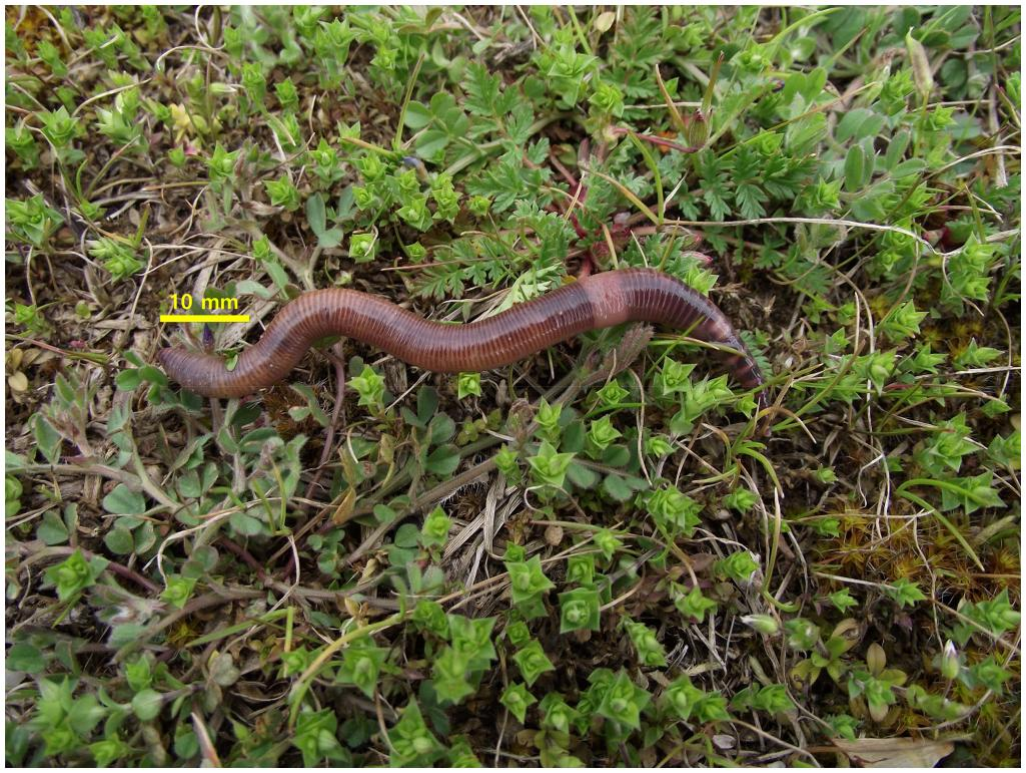
Clitellate specimen of *Dendrobaena veneta*. The photograph was taken in Piliscsaba, Hungary by the first author Csaba Csuzdi on 20 June 2022.

The genus *Dendrobaena* Eisen, 1873 is one of the most speciose genera in the family Lumbricidae. *Dendrobaena veneta* (Rosa, 1886) is a widely distributed peregrine species, probably of East Mediterranean origin (Szederjesi et al. 2019; Figure 1). As it is one of the earthworms suitable for vermicomposting this species is established all over Europe, where it is mainly found in compost and manure heaps and other disturbed habitats rich in organic matter. *D. veneta* is a medium-sized worm with vivid red–violet stripes and the following morphological characteristics: length 30–110 mm, diameter 4–8 mm, and 54–150 segments. Clitellum on 26, 27–32, 33, tubercles on 30–31. Spermathecal pores in 9/10, 10/11 near M (Csuzdi and Zicsi 2003). It is closest to its sister species *Dendrobaena succinta* (Rosa, 1905) distributed in natural habitats of the East Mediterranean but easy to distinguish because *D. succinta* has a uniform red coloration and lacks the characteristic stripes of *D. veneta* (Szederjesi et al. 2019).

## Materials

### Samples

The specimens of *D. veneta* were collected in Albania, Tiranë district, Gropë Mts, Bizë, Kaprol stream, and its sidebrook at the military camp (41°33′92″N, 20°19′89″E; 1250 m) on 7 July 2019 (T. Szederjesi coll). The specimen was identified morphologically according to the characters detailed in Szederjesi et al. (2019) and corroborated by using its COI barcode as well. The specimen is deposited at Jeonbuk National University (Yong Hong, yonghong@jbnu.ac.kr) under the voucher number JBNU0023.

## Methods

### Sequencing, assembling, and annotation

Total DNA was isolated from single specimen of *D. veneta*. A sequencing library was constructed using the Illumina TruSeq DNA Nano Library Prep Kit (Illumina Inc., San Diego, CA, USA). The mitogenome sequence was determined using the Illumina HiSeq-X platform (San Diego, CA, USA). The raw reads were *de novo* assembled using SPAdes version 3.13.0 (Bankevich et al. 2012) and NOVOplasty version 4.3.1. The depth of coverage was determined by Geneious prime implemented with Bowtine2 by mapping the total reads to the newly assembled sequence (Figure S1). The resulting complete sequences were used for the mitogenome annotation using MITOS webserver (Donath et al. 2019) and manually curated based on BLAST searches in the National Center for Biotechnology Information (NCBI) database.

### Phylogenetic analysis

Phylogenetic analysis was conducted using 26 earthworm (23 Lumbricidae and 3 Megascolecidae) mitogenome sequences including *D. veneta*. These include the publicly available complete mitogenome sequences of 23 Lumbricidae species and 3 representative Megascolecidae species as outgroups. Some mitogenome sequences (CM035405, MZ857197, MZ857199, MZ857200, MZ857201) were annotated using MITOS webserver and manually curated. Although the mitogenome of *D. tellermanica* is not complete, it contained the entire set of 13 protein-coding genes (PCGs); therefore, it was included in the analysis as well. Nucleotide sequences of 13 PCGs from each mitogenome were extracted using Geneious Prime 2023. Each gene was aligned by the MAFFT version 7 (Katoh and Standley 2013). Gaps and ambiguous sites in the alignments were then trimmed using trimAl version 2.3 (Capella-Gutiérrez et al. 2009). Geneious Prime 2023 was used to concatenate individual gene alignments into a dataset. Substitution saturation for the 13 PCGs dataset was assessed by Xia’s test and index of substitution saturation (Iss) with a GTR model as implemented in DAMBE v7.3.32 (Xia 2018). The result revealed that the 13 PCGs were not saturated, with the value of the Iss obviously lower than the critical Iss value. Best-fitting nucleotide substitution model was selected on the basis of the Bayesian information criterion with ModelFinder implemented in IQ-TREE v 2.1.3 (Nguyen et al. 2015). An ultrafast bootstrap maximum-likelihood tree was created with IQ-TREE with 5000 bootstrap replicates using the GTR + F + I + G4 substitution model. Bayesian inference phylogeny was inferred using MrBayes v3.2.7a (Ronquist et al. 2012) under the GTR + I + G + F model (2 parallel runs, 1,000,000 generations), in which the initial 25% of sampled data were discarded as burn-in. Convergence was assessed by parameters including effective sample sizes (ESS) and average standard deviation of split frequencies (ASDSF). The values (ESS > 200 and ASDSF < 0.01) were considered adequate.

## Results

**Figure 2.**
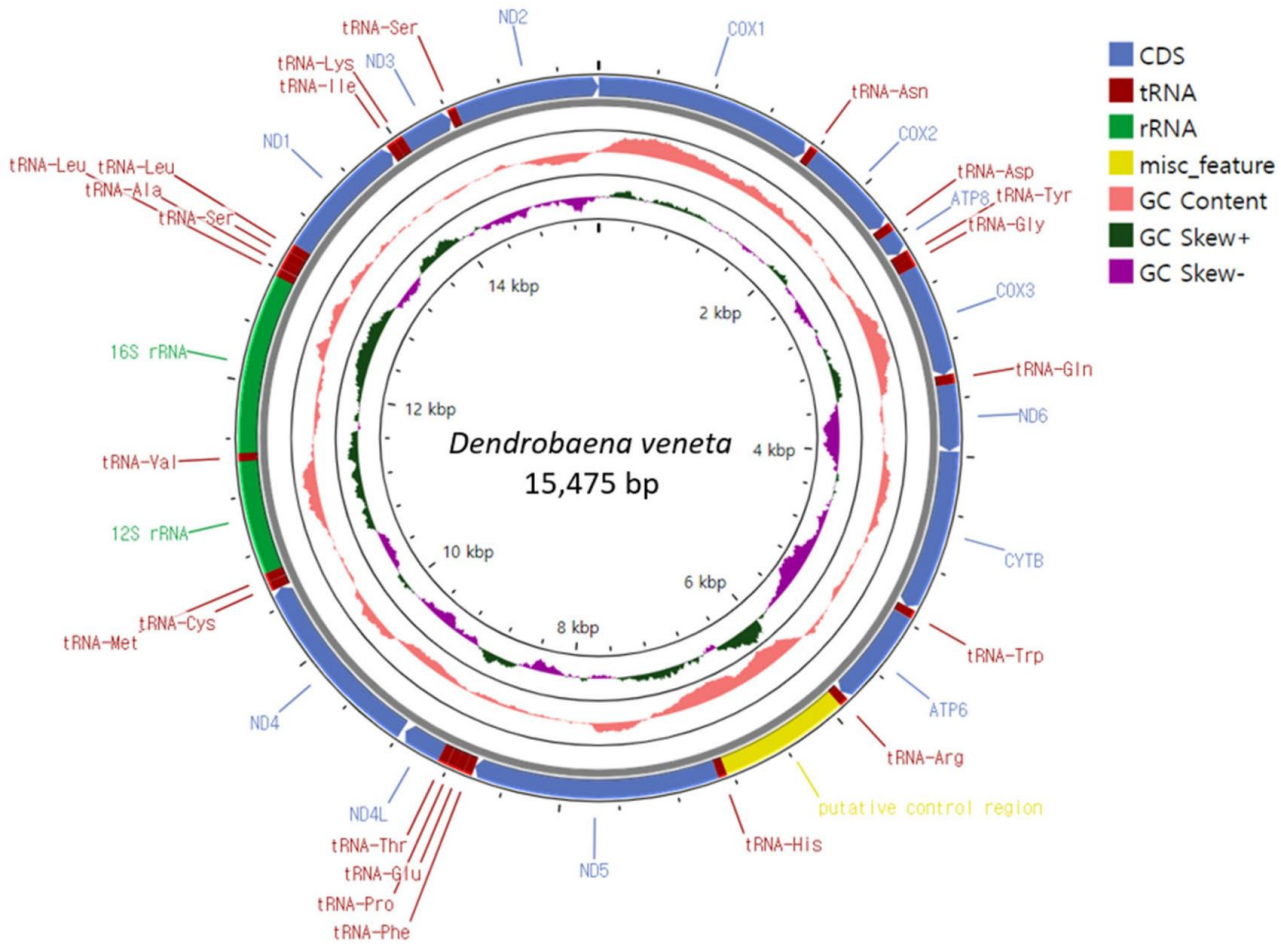
Circular sketch map of the *Dendrobaena veneta* mitogenome. The mitogenome map was generated using the GenBank format of the sequence (OQ763213) with the online Proksee software (Grant et al. 2023). From the inside to the outside, the innermost scale indicates the length, the inner circle indicates the GC skew, the Middle circle indicates the GC content, and the outer circle indicates gene arrangement. The plots of the GC content and GC skew were generated by default setting of the software (window 500, step 1). different colors represent different gene blocks.

**Figure 3.**
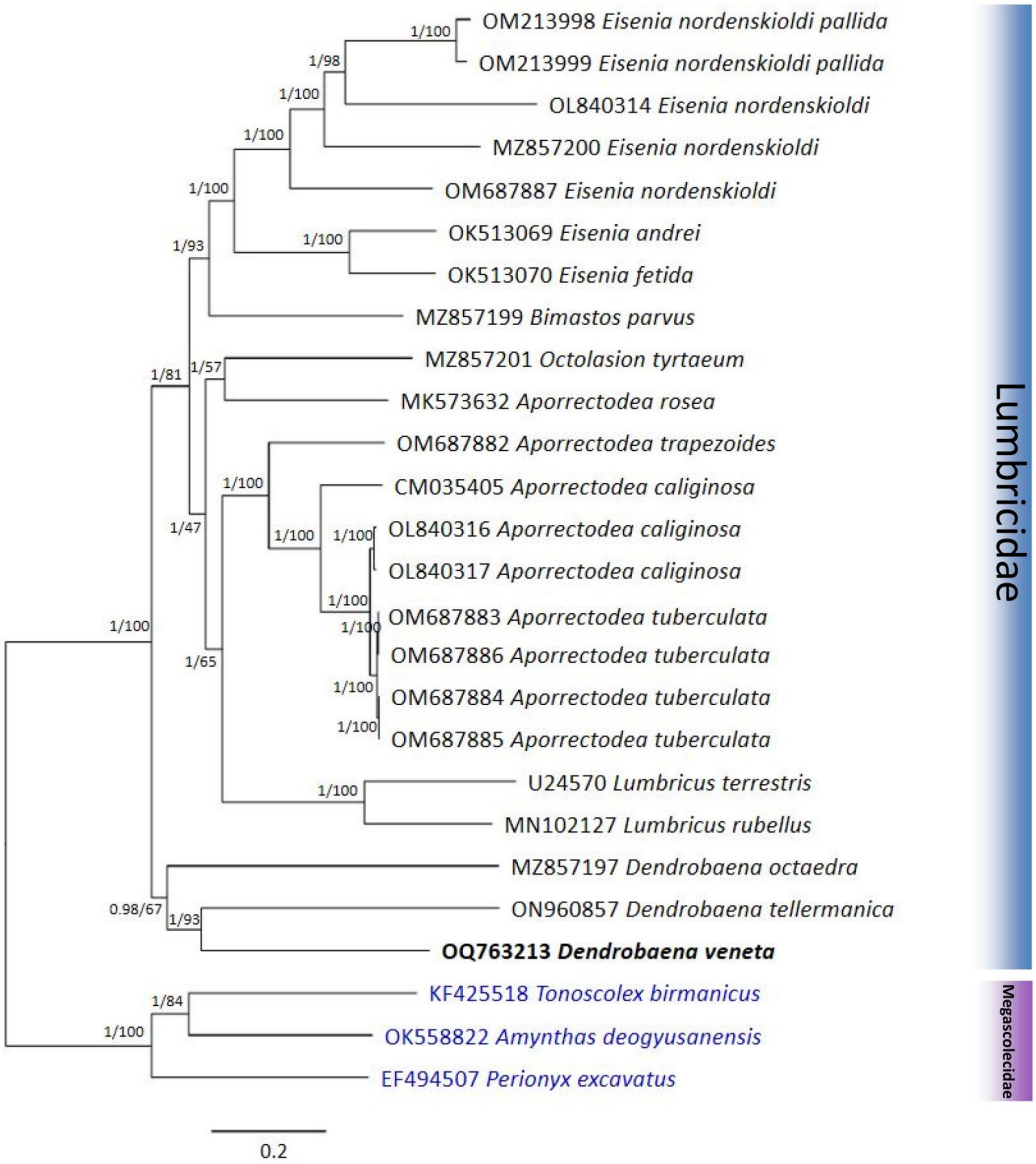
Phylogenetic tree of the 26 species of the Megascolecidae and Lumbricidae. Phylogenetic trees were reconstructed using maximum-likelihood (ML) and Bayesian inference (BI) methods, based on the nucleotide sequence of 13 PCGs. The sequences used in the tree are listed in Table 1. The numbers at each node specify the BI posterior probability and ultrafast bootstrap support (%), respectively. The scale bar indicates the number of substitutions per site.

The complete mitogenome of *D. veneta* is a circular DNA molecule consisting of 15,475 bp. Its structure is typical for earthworms with 13 PCGs, 22 tRNAs, and 2 rRNAs (Figure 2) located on the heavy strand. We observed that all 13 PCGs start with an ATG codon. Of these, 10 PCGs end with complete stop codons, TAA and TAG, and 3 PCGs end with an incomplete stop codon, T. The A + T content of the whole mitogenome is 61.2%. The sequencing reads were mapped to the assembled mitogenome sequences to evaluate the coverage of depth. At present, only a few complete Lumbricidae mitochondrial genome (mitogenome) sequences are available. Two mitogenome sequences have been reported to date, especially in the genus *Dendrobaena*, including a complete sequence of *D. octaedra* and a near complete sequence of *D. tellermanica. D. veneta* in the maximum-likelihood and Bayesian inference trees grouped together with the other two *Dendrobaena* species (Figure 3), and the positions of the other Lumbricidae species are similar to the previous studies (Csuzdi et al. 2022; Zhao et al. 2022; Csuzdi et al. 2023; Shekhovtsov et al. 2023). The newly analyzed *D. veneta* formed a well-supported clade together with the other two *Dendrobaena* species including *D. octaedra* the type of the genus. Not surprisingly, *D. veneta* seems to be most close to the Caucasian *D. tellermanica* which corroborates the Eastern Mediterranean origin of *D. veneta* (Perel 1979; Szederjesi et al. 2019).

**Table 1.** List of metagynophora mitogenomes used in this study.

Species	Genbank No.	Genome	Reference
*Eisenia nordenskioldi pallida*	OM213998 OM213999	Complete	Csuzdi et al. 2023
*Eisenia nordenskioldi*	OL840314 MZ857200 OM687887	Complete	Zhao et al. 2022
*Eisenia andrei*	OK513069	Complete	Csuzdi et al. 2022
*Eisenia fetida*	OK513070	Complete	Csuzdi et al. 2022
*Bimastos parvus*	MZ857199	Complete	Liu et al. 2021
*Octolasion tyrtaeum*	MZ857201	Complete	Liu et al. 2021
*Aporrectodea rosea*	MK573632	Complete	Shekhovtsov and Peltek 2019
*Aporrectodea trapezoides*	OM687882	Complete	Unpublished
*Aporrectodea caliginosa*	CM035405	Complete	Unpublished
*Aporrectodea caliginosa*	OL840316 OL840317	Complete	Zhao et al. 2022
*Aporrectodea tuberculata*	OM687883 OM687884 OM687885 OM687886	Complete	Zhao et al. 2022
*Lumbricus terrestris*	U24570	Complete	Boore and Brown 1995
*Lumbricus rubellus*	MN102127	Complete	Zhang et al. 2019
*Dendrobaena octaedra*	MZ857197	Complete	Liu et al. 2021
*Dendrobaena tellermanica*	ON960857	Partial	Shekhovtsov et al. 2023
*Dendrobaena veneta*	OQ763213	Complete	This study
*Tonoscolex birmanicus*	KF425518	Complete	Wang et al. 2015
*Amynthas deogyusanensis*	OK558822	Complete	Koo and Hong 2023
*Perionyx excavatus*	EF494507	Complete	Unpublished

## Discussion and conclusions

Morphologically the genus *Dendrobaena* is quite heterogeneous especially because it contains species with fasciculate and pinnate types of longitudinal musculature (Csuzdi and Zicsi 2003) a character which was once thought to be of high importance in earthworm phylogeny (Pop 1941; Omodeo 1956; Zicsi 1985). However, previous mitogenome analyses have shown that this character is highly homoplasious (Shekhovtsov et al. 2020; Csuzdi et al. 2022). This observation is supported by our present studies as well, since *D. veneta* has fasciculate but *D. octaedra* possesses pinnate musculature.

However, our knowledge on Oligochaeta, especially Lumbricidae mitogenomes is still limited. More mitogenome sequences of Oligochaeta species would be helpful in creating more robust mitogenome-based phylogeny and for further understanding of Oligochaeta mitogenome evolution (Boore and Brown 1995).

## Supplementary Material

Supplemental Material

## Data Availability

The genome sequence data that support the findings of this study are openly available in GenBank of NCBI under accession No. OQ763213. The associated BioProject, SRA, and Bio-Sample numbers are PRJNA769829, SRR24457999, and SAMN34844758, respectively.
