# Supplementary material for: Complete mitochondrial genome of the composting worm Dendrobaena veneta (Clitellata: Oligochaeta, Lumbricidae)
Source: Mitochondrial DNA B Resour. 2023 Oct 13;8(10):1082–6. doi: 10.1080/23802359.2023.2265177 (PMC10578090; doi:10.1080/23802359.2023.2265177)
Supplement: Supplemental Material [file TMDN_A_2265177_SM2800.docx]

**Supplementary data**

**Complete mitochondrial genome of the composting worm *Dendrobaena veneta* (Clitellata: Oligochaeta, Lumbricidae)**

Csaba Csuzdi^1a^, Jachoon Koo^2a^, Nakjung Choi^3^, Tímea Szederjesi^4^ and Yong Hong^5b^

^1^Department of Zoology, Eszterházi Károly Catholic University, Eger, Hungary; [csuzdi.csaba@uni-eszterhazy.hu](mailto:csuzdi.csaba@ektf.hu)

^2^Division of Science Education and Institute of Fusion Science, College of Education, Jeonbuk National University, Jeonju 54896, Korea; [jkoo@jbnu.ac.kr](mailto:jkoo@jbnu.ac.kr)

^3^National Institute of Crop Science, Wanju-gun, Korea; [njchoi@korea.kr](mailto:njchoi@korea.kr)

^4^Department of Systematic Zoology, Eötvös Loránd University, Budapest, Hungary; t.szederjesi@gmail.com

^5^Department of Agricultural Biology, College of Agriculture & Life Sciences, Jeonbuk National University, Jeonju, Republic of Korea; yonghong@jbnu.ac.kr

^a^ These authors contributed equally.

^b^ Correspondence:

Yong Hong

Tel: +82-63-270-2529, Fax: +82-63-270-2531, e-mail: yonghong@jbnu.ac.kr


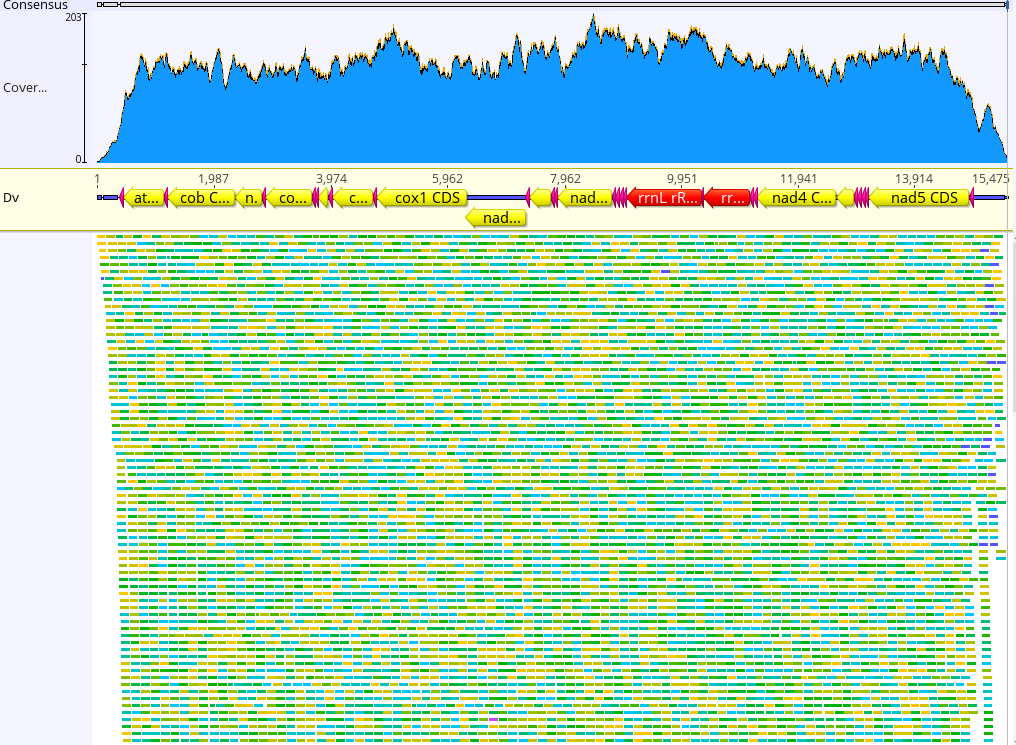


Supplementary Figure S1. Coverage plot of mitogenome of *Dendrobaena veneta*. Among 166,854 reads, the 97,533 reads were mapped to an assembly result (100% of coverage, 147.94 of average mapping depth) using Bowtie2 in Genius prime (ver. 2023.0.2).
